# Autophagy regulation is an effective strategy to improve the prognosis of chemically induced acute liver injury based on experimental studies

**DOI:** 10.1111/jcmm.15565

**Published:** 2020-07-06

**Authors:** Chenxia Hu, Lingfei Zhao, Miaoda Shen, Zhongwen Wu, Lanjuan Li

**Affiliations:** ^1^ Collaborative Innovation Center for the Diagnosis and Treatment of Infectious Diseases State Key Laboratory for the Diagnosis and Treatment of Infectious Diseases The First Affiliated Hospital School of Medicine Zhejiang University Hangzhou PR China; ^2^ National Clinical Research Center for Infectious Diseases The First Affiliated Hospital School of Medicine Zhejiang University Hangzhou PR China; ^3^ Key Laboratory of Kidney Disease Prevention and Control Technology Kidney Disease Center Institute of Nephrology First Affiliated Hospital College of Medicine Zhejiang University Hangzhou PR China; ^4^ Department of Orthopedics The First Affiliated Hospital School of Medicine Zhejiang University Hangzhou PR China

**Keywords:** acute liver injury, autophagy, liver regeneration, mechanism, mesenchymal stem cell

## Abstract

Acute liver injury (ALI) induced by chemicals in current experimental studies is characterized by inflammation, oxidative stress and necrosis, which can greatly influence the long‐term outcome and lead to liver failure. In liver cells, different autophagy forms envelop cytoplasm components, including proteins, endoplasmic reticulum (ER), mitochondria and lipids, and they effectively participate in breaking down the cargo enclosed inside lysosomes to replenish cellular energy and contents. In general, autophagy serves as a cell survival mechanism in stressful microenvironments, but it also serves as a destructive mechanism that results in cell death in vitro and in vivo. In experimental animals, multiple chemicals are used to mimic ALI in patients to clarify the potential pathological mechanisms and develop effective strategies in the clinic. In this review, we summarize related publications about autophagy modulation to attenuate chemically induced ALI in vitro and in vivo. We also analysed the underlying mechanisms of autophagy regulators and genetic modifications to clarify how to control autophagy to protect against chemically induced ALI in animal models. We anticipate that selectively controlling the dual effects of hepatic autophagy will help to protect against ALI in various animals, but the detailed mechanisms and effects should be determined further in future studies. In this way, we are more confident that modulating autophagy in liver regeneration can improve the prognosis of ALI.

## 1 INTRODUCTION

**FIGURE 1:**
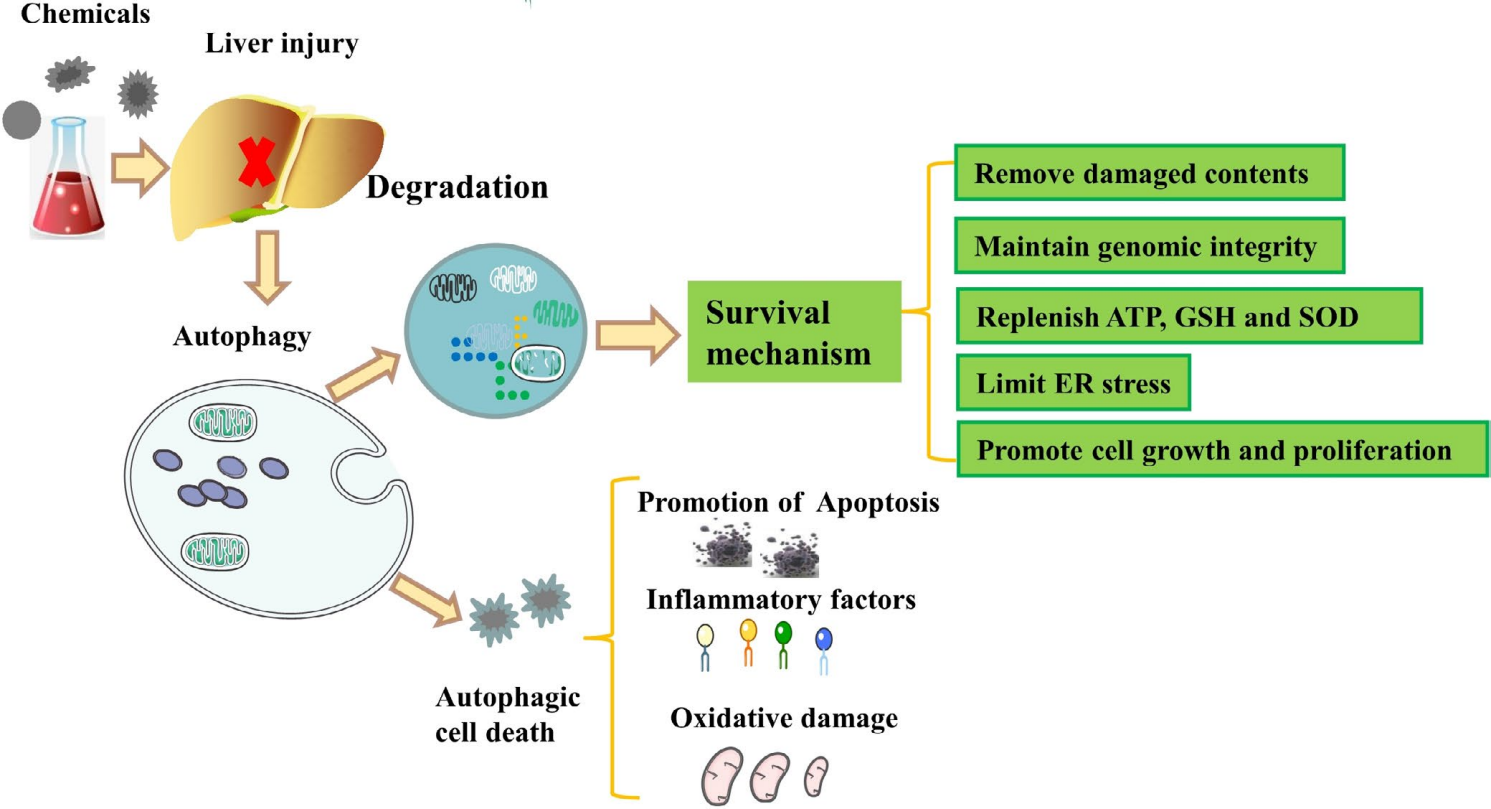
Autophagy is a survival mechanism or a cell death mechanism in liver injury

Although most liver cells are quiescent under normal conditions, liver regeneration is initiated after partial hepatectomy to compensate for liver function. The liver is also a digestive organ that is always exposed to orally ingested antigens and harmful products from intestinal bacteria since liver tissue is surrounded by systemic blood circulation from portal blood. In response to liver injury, liver‐specific apoptosis and autophagy occur simultaneously but act independently through different pathways, influencing each other and participating in the initiation of liver injury and liver regeneration. Acute liver injury (ALI) is characterized by inflammation, oxidative stress and necrosis, which can greatly influence the long‐term outcome and lead to liver failure. Autophagy, a form of cell death marked by partial chromatin condensation is considered to be upstream of apoptosis and occurs earlier than apoptosis. On the other hand, apoptosis is a form of cell death marked by DNA fragmentation and is the terminal form of cell death. Apoptosis is characterized by nuclear fragmentation, chromatin condensation and cellular shrinkage. It acts as a scavenger to maintain tissue homeostasis during the development and ageing of tissues after stimulation by immune reactions, diseases or noxious agents.^1^ Misfolded proteins, lipid deposition and damaged mitochondria accumulate in vivo under pathological states, while protective autophagy has been proven to promote cell survival and maintain cell activity by clearing these adverse factors.^2^ Multiple studies have attempted to clarify the related mechanisms of autophagy in the attenuation of apoptosis as follows: (a) autophagy is able to clear damaged organelles, cell debris, external pathogens or denatured subcellular constituents^3^; (b) although mutagenic microenvironments continuously stimulate gene mutations in mammals, autophagy effectively maintains the genomic integrity of cells or tissues^4^; (c) autophagy is also able to supply more energy and maintain cell or tissue homeostasis in mammals by degrading cytosolic components in lysosomes^5^; (d) autophagy significantly degrades unfolded protein aggregates and inhibits endoplasmic reticulum (ER) stress to maintain ER function^6^; and (e) autophagy promotes cell growth and proliferation in local injured tissues.^7^ As a result, activation of apoptotic pathways further promotes the inactivation of autophagy. BCL2‐interacting protein 3 (BNIP3) can significantly increase the apoptosis rate by sequestering B‐cell lymphoma protein‐2 (Bcl‐2) family proteins and reducing the binding between Bcl‐2 and Beclin‐1.^8^ Activation of apoptosis further cleaves and inactivates Beclin‐1, resulting in suppression of autophagy in a caspase‐dependent manner.^9^ Moreover, autophagy improves the survival rate of quiescent hepatocytes by continually recycling nucleic acids, complex carbohydrates, lipids and proteins in liver tissue. It is worth noting that excessive accumulation of autophagic factors will ultimately result in cell death after disruption of the adaptive mechanism under extremely harmful conditions.^10^ The final products of autophagy contain small sealed membrane vesicles similar to apoptosis, but autophagic cell death significantly increased the contents of autophagosomes and autolysosomes in vivo.^11^, ^12^ It is generally anticipated that impairment of lysosomal activities would result in autophagosome deposition, cellular dysfunction and activation of caspase‐dependent cell death.^13^ On the other hand, purified Beclin‐1‐C is generated in a caspase‐dependent manner and effectively promotes the generation of mitochondrial cytochrome c to stimulate apoptosis.^14^ Remarkably, autophagy is a homeostatic response that helps to clear damaged cells and hepatotoxic factors in vivo, but it also results in a stressful microenvironment and tissue injury (Figure 1).

As liver cell apoptosis is a prominent pathological process during severe liver injury, interventions on autophagy in liver tissue help to regenerate liver tissue and inhibit cellular apoptosis. Pharmacological autophagy modulators or genetic modifications may provide protective effects in liver tissue via their hepatoprotective and anti‐inflammatory effects. In this review, we summarize related publications about autophagy modulation for attenuating ALI in vitro and in vivo. We also analysed the underlying mechanisms of autophagy regulators and genetic modifications to clarify how to control autophagy to protect against chemically induced ALI in animal models. We conclude that strategies targeting the regulation of autophagic flux in liver tissue will further improve the prognosis of patients with ALI.

## 2 CURRENT FORMS OF AUTOPHAGY IN LIVER TISSUE

**FIGURE 2:**
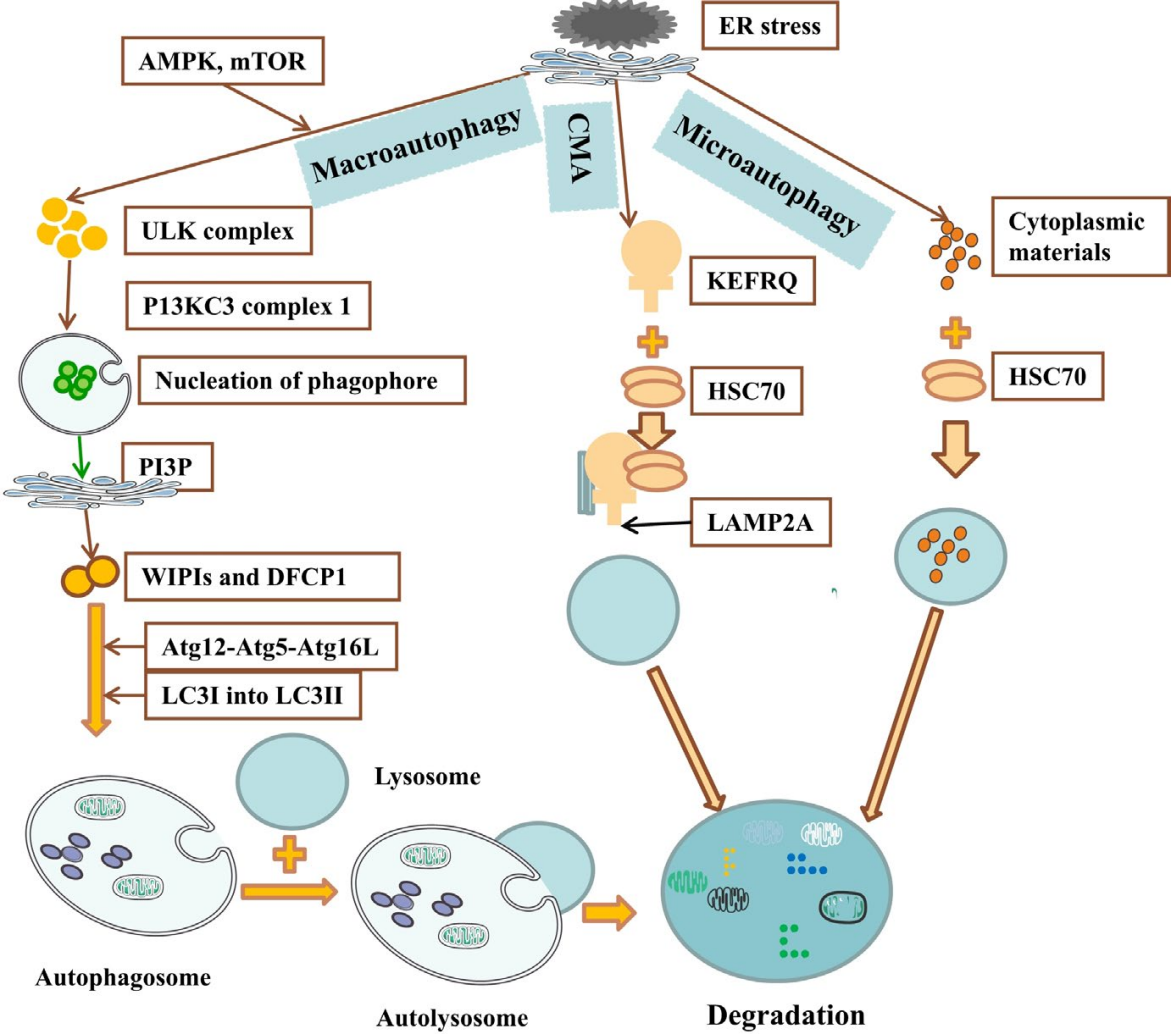
The detailed mechanisms of macroautophagy, chaperone‐mediated autophagy and microautophagy in liver tissue are shown

Macroautophagy, chaperone‐mediated autophagy (CMA) and microautophagy are the three main types of autophagy in mammals (Figure 2). The complete process of macroautophagy includes six steps: initiation, nucleation, elongation, closure, maturation and degradation.^15^, ^16^, ^17^ Recognition of ER stress activates mechanistic target of rapamycin kinase (mTOR) and AMP‐activated protein kinase (AMPK) for activation of unc‐51‐like kinase (ULK) protein and subsequent activation of phosphatidylinositol‐3‐phosphate (PI3P) in the ER membrane. Although the generation of the omegasome is not indispensable for autophagosome formation, double FYVE‐containing protein 1 (DFCP1) is an effector protein for omegasome formation and subsequent autophagosome generation.^18^ After synthesis of PI3P from the nascent phagophore, the mammalian effector protein WD repeat domain phosphoinositide‐interacting protein (WIPI) recognizes this protein.^19^ The PI3P‐binding protein complex contains WIPI, and autophagy‐related (Atg)2 accumulates on the isolation membrane and contributes to expansion.^18^ PI3P effectively recruits Atg12‐Atg5‐Atg16L and promotes the conversion of microtubule‐associated protein light chain 3 (LC3)I into LC3II. After that, cytoplasmic materials are sequestered by the phagophore (a preautophagosomal membrane structure), which thereafter expands and encloses its cargo to form an autophagosome (a double‐membrane vesicle).^16^, ^20^ Then, these autophagosomes fuse into lysosomes and generate autolysosomes for degradation of the enclosed cargo by acid hydrolases and recycling into biologically active monomers to maintain cellular metabolic homeostasis.^20^ CMA is a form of autophagy that uniquely and selectively degrades abundant substrate proteins by delivering them into the lysosome one by one.^21^ Heat shock cognate (HSC)70 recognizes a specific cytosolic protein that contains a KFERQ‐like pentapeptide and subsequently interacts with lysosomal‐associated membrane protein 2 (LAMP2A) to degrade cellular contents.^16^ Under stress conditions, microautophagy effectively maintains organelles and membrane homeostasis by degrading cytoplasmic contents in lysosomes through invagination or deformation of the lysosomal membrane.^22^ The liver is also a large and special organ that has abundant mitochondria and is an important site for the metabolization of glucose and fat storage. In consideration of this, other selective autophagy processes, such as mitophagy and lipophagy, were also found to exist in cultured hepatocytes and liver tissue. Mitophagy is a kind of autophagy that effectively removes damaged mitochondria to reduce mitochondria‐derived reactive oxygen species (ROS) and cell death factors in vivo.^3^, ^23^ Lipophagy selectively degrades hepatic triglycerides (TG) by genetic and chemical manipulation of the autophagic pathway, while knockdown of Atg5 or Atg7 significantly up‐regulates the levels of hepatic TG in fatty acid–treated hepatocytes or high‐fat‐fed mice.^24^ In addition, chloroquine (a lysosomal inhibitor) significantly suppresses the fusion between autophagosomes and lysosomes and the degradation of lysosomes, subsequently improving the accumulation of TG in fatty acid‐treated hepatocytes.^25^, ^26^ Activated lysosomal enzymes degrade all discarded cellular contents, such as those from the cytoplasm, mitochondria, ER, peroxisomes, Golgi apparatus and other organelles.

## 3 AUTOPHAGY AND CHEMICALLY INDUCED ALI IN ANIMAL MODELS

To mimic ALI in patients, multiple chemicals, such as acetaminophen (APAP), d‐galactosamine (D‐GalN), lipopolysaccharide (LPS), concanavalin A (ConA), carbon tetrachloride (CCl_4_) and other chemicals, have been used to generate ALI animal models. According to current studies, each chemical triggers ALI via different mechanisms. Autophagy regulation is reported to exert protective effects in these models via various mechanisms (Table 1).

**TABLE 1:** The modulation of autophagy effectively protects animal models from liver injury induced by various hepatotoxic factors via multiple mechanisms

Animal	Chemical	Treatment	Effect on autophagy	Effect	Other mechanism	Ref
GFP‐LC3 transgenic mice	APAP	Rapamycin	↑	Decrease centrilobular necrosis	Replenish GSH	31
Mice	APAP	IL‐22	↑	Decrease serum levels of ALT and AST; maintain liver histology	Alleviate hepatic oxidative stress; reduce the expression of hepatic inflammatory factors; no alteration of hepatic APAP metabolism	34
Mice	APAP	ALR	↑	Attenuate the up‐regulation of ALT, AST, MPO, MDA and ROS; decrease intrahepatic haemorrhage and necrosis; decrease SOD and GSH depletion; decrease the release of AIF and cytochrome c; decrease apoptosis	Up‐regulate SOD and GSH; inhibit MPO, MDA, ROS, intrahepatic haemorrhage and necrosis	35
Mice	APAP	Chlorpromazine	↑	Down‐regulate ALT level; decrease necrosis rate of hepatocytes	Inactivate JNK pathway; no effect on the metabolic activation of APAP	36
Mice	APAP	Glycycoumarin	↑	Decrease ALT level and hepatic necrosis	Activate JNK signalling pathway; no activation of the Nrf2 signalling pathway	38
Mice	APAP	Alpha‐mangostin	↓	Improve histopathological changes; decrease the levels of ALT and AST	Decrease the release of pro‐inflammatory factors including TNF‐α and IL‐1β; increase the expression of Bcl‐2; decrease the expression of Bax and cleaved caspase 3; decrease oxidative stress	39
Mice	D‐GalN/LPS	AMPK activator AICAR	↑	Decrease ALT and AST levels	Enhance the expression of PPAR‐α; abrogate the effect of miR‐19a mimic	43
Mice	D‐GalN/LPS	FK866 or rapamycin	↑	Decrease ALT and AST levels; maintain liver histology	Suppress the JNK pathway	44
Mice	ConA	FK866 or rapamycin	↑	Decrease ALT and AST levels; maintain liver histology	Suppress the JNK pathway	44
Mice	D‐GalN/LPS	Wy‐14 643	↑	Protect D‐GalN/LPS‐induced ALF mice against liver injury	Up‐regulate PPAR‐α expression; suppress inflammation; inhibit NF‐κB p65, JNK and ERK pathways	45
Mice	D‐GalN/LPS	ACY1215	↑	Maintain normal liver histology and function	Inhibit apoptosis	46
Rat	D‐GalN/LPS	P13K inhibitor	↑	Maintain liver function	Inhibit the PI3K/AKT/mTOR signalling pathway	47
Mice	D‐GalN/LPS	Tectorigenin	↑	Decrease the mortality of mice; decrease necrosis and severe tissue haemorrhage	Reduce TLR4 expression; inhibit MAPK and NF‐κB pathways; suppress the secretion of TNF‐α and IL‐6.	48
Mice	D‐GalN/LPS	Daphnetin	↑	Decrease lethality in mice, decrease ALT and AST levels	Down‐regulate the levels of TNF‐α, IL‐1β, IL‐6, MDA and MPO, iNOS and COX‐2; up‐regulate the levels of GSH and SOD; inhibit NLRP3; activate MAPK and NF‐κB pathways	49
Mice	D‐GalN/LPS	MSCs	↑	Improve survival rate, liver function, histology; decrease Ki67 and TUNEL	Up‐regulate HO‐1 expression; decrease inflammatory factors	50
Mice	ConA	3‐MA and bafilomycin A1	↓	Inhibit the maturation of DCs	Decrease expression of inflammatory factors	54
Mice	ConA	Methylprednisolone	↓	Inhibit apoptosis and autophagy in hepatocytes	Down‐regulate inflammatory reactions; activate the Akt/mTOR signalling pathway	55
Mice	ConA	Necrostatin‐1	↓	Preserve liver functions; maintain normal histopathology	Suppress secretion of inflammatory cytokines (TNF‐ α, IFN‐γ, IL2, IL6, and cobalt ion binding protein)	56
Mice	ConA	15d‐PGJ2	↓	Preserve liver functions and normal histopathology	Inhibit mitochondrial ROS and release of inflammatory factors (TNF‐α, IL‐1β); up‐regulate the antioxidative stress factor HO‐1	57
Mice	ConA	Oleanolic acid	↓	Decrease the levels of serum liver enzymes and inflammatory factors	Improve the expression of PPAR‐α; inhibit the phosphorylation of JNK	58
Mice	ConA	Astaxanthin	↓	Decrease serum levels of liver enzymes	Down‐regulate JNK/p‐JNK‐mediated apoptosis and autophagy‐related pathways	59
Mice	ConA	Shikonin	↓	Preserve liver functions; maintain normal histopathology	Down‐regulate release of inflammatory factors such as IL‐1β, TNF‐α, and IFN‐γ; inhibit the JNK pathway	60
Mice	ConA	Quercetin	↓	Decrease the expression levels of liver enzymes and inflammatory cytokines	Attenuate the expression of Bax/Bcl‐2 and caspase‐9	61
Mice	ConA	Isorhamnetin	↓	Decrease the serum levels of liver enzymes and inflammatory cytokines; improve pathological damage	Inhibition of apoptosis and autophagy via the P38/PPAR‐α pathway	62
Mice	ConA	Fucosterol	↓	Preserve liver functions and attenuate liver necrosis and apoptosis	Down‐regulate the release of TNF‐α, IL‐6, and IL‐1β; down‐regulate Bax/Bcl‐2; inhibit P38 MAPK/PPARγ/NF‐κB signalling	63
Mice	ConA	Salidroside	↓	Reduce the expression of liver enzymes and attenuate pathological damage	Attenuate inflammation and the PI3K/AKT signalling pathway	64
Mice	CCl_4_	Genipin	↑	Ameliorate histological liver changes; decrease ALT and AST levels	Down‐regulate NF‐κB and STAT3‐dependent inflammation	67
Mice	CCl_4_	Chloroquine	↓	Decrease ALT, AST and histological changes	Down‐regulate NF‐κB, p53, the ratio of Bax/Bcl‐2, and caspase‐3 in liver tissue; attenuate the secretion of HMGB1‐mediated inflammatory factors	68
Atg7 knockout mice	APAP	Cyclosporine A or JNK inhibitor	↑	Improve liver function	Decrease ROS levels and mitochondrial damage; increase the expression of caspase‐3 and caspase‐7	69
Atg5 knockout Mice	APAP	N/A	↓	Increase hepatocyte proliferation; maintain histological and clinical chemistry parameters	Activate NRF2 pathway; promote hepatocyte proliferation	72
Mice	CCl_4_	ASPP2	↑	Decrease ALT and AST levels; decrease hepatic tissue haemorrhage and necrosis	Reduce cellular inflammation and apoptosis	74

### 3.1 Modulation of autophagy reduces APAP‐induced liver injury

The liver is the largest organ for the metabolization of drugs and detoxification of toxins, and frequent exposure to toxic drugs leads to a high prevalence of liver failure. Drug‐induced ALI commonly targets mitochondria and then induces cell death; thus, modulation of mitophagy will be a major target for improving cell survival. In the clinic, the intake of various drugs, such as antipyretics, chemotherapeutics and antivirals, induces ALI. Overdose of APAP, a commonly used antipyretic and analgesic drug, easily triggers ALI in mammals.^27^ Most patients with APAP overdose progress into acute liver failure (ALF) and do not receive medical care until they are past the metabolic phase, which results in the high mortality of APAP overdose–induced liver injury in the clinic. Glucuronic acid and sulphate conjugation of APAP promote the secretion of APAP into the bile or blood in the liver. The conversion of APAP into N‐acetyl‐p‐benzoquinone imine (NAPQI) by cytochrome P450 further promoted the depletion of liver stores of glutathione (GSH) and induced the disturbance of cellular redox homeostasis.^28^ APAP enhances the posttranslational modifications of Parkin by increasing nitric oxide (NO) and reactive nitrogen species to generate mitochondrial spheroids.^29^ APAP overdose results in severe liver damage after the induction of mitochondrial dysfunction, oxidative stress and mitogen‐activated protein kinase (MAPK) activation.^30^ In addition, APAP promotes the abnormal accumulation of protein adducts in mitochondria and induces mitochondrial damage, caspase‐independent cell death and necrosis, which further activate the autophagic response to remove damaged mitochondria and prevent APAP‐induced injury.^31^ Wang et al demonstrated that APAP overdose effectively activated mitophagy to degrade damaged mitochondria and mitochondrial proteins in primary mouse hepatocytes, indicating that modulation of mitophagy may play a role in maintaining homeostasis of hepatic metabolism.^32^ Although multiple apoptosis‐related pathways are activated after hepatocytes or animals are treated with APAP, the expression of Krüppel‐like factor 6 (KLF6) is also increased to activate autophagy to promote liver regeneration in a p53‐dependent manner.^33^

In consideration of current evidence, the activation of autophagy may serve as a potential treatment for APAP hepatotoxicity, and multiple drugs have been used to protect against APAP‐induced ALI in recent studies. Rapamycin significantly enhanced the autophagy process and decreased APAP‐induced cell death in cultured primary hepatocytes and in mouse liver, although it was not effective in abrogating APAP‐induced GSH depletion.^31^ Interleukin (IL)‐22 treatment significantly alleviated APAP‐induced cytotoxicity via up‐regulation of hepatic LC3II and p‐AMPK, as demonstrated by down‐regulation of serum aminotransferases, liver ROS and liver necrosis in vitro and in vivo.^34^ Augmenter of liver regeneration (ALR) attenuated APAP‐stimulated alterations via up‐regulation of autophagosomes, LC3I to LC3II conversion, p62 degradation and the levels of superoxide dismutase (SOD) and GSH but inhibition of myeloperoxidase (MPO), malondialdehyde (MDA), ROS, intrahepatic haemorrhage and necrosis.^35^ Cotreatment or post‐treatment with chlorpromazine (CPZ), a dopamine inhibitor with an antischizophrenia effect, down‐regulated the alanine transaminase (ALT) level and necrosis rate of hepatocytes in APAP‐treated mice via up‐regulation of autophagy and inactivation of the c‐Jun‐N‐terminal kinase (JNK) pathway without affecting the metabolic activation of APAP.^36^

In addition, a number of plant ingredients also participate in repairing liver damage via modulation of autophagy. Dihydroquercetin reversed all the changes induced by APAP via activation of autophagy and phosphorylation of the Janus kinase 2/signal transducer and activator of transcription 3 (JAK2/STAT3) cascade, which subsequently inhibited ROS accumulation, mitochondrial dysfunction, extracellular signal regulated kinase‐JNK stress and necrosis.^37^ Glycycoumarin, which is purified from licorice, is able to alleviate APAP‐induced oxidative stress and liver injury via activation of protective autophagy and the JNK signalling pathway but not via activation of the nuclear factor erythroid 2 like 2 (NRF2) signalling pathway.^38^ However, another study showed that pre‐treatment with alpha‐mangostin partly inhibited the activation of autophagic cell death via down‐regulation of p‐mTOR, p‐AKT and the LC3II/LC3I ratio, subsequently decreasing the release of inflammatory factors, including tumour necrosis factor (TNF)‐α and IL‐1β, and inhibiting the activation of apoptotic pathways in APAP‐induced ALI models.^39^ Related studies indicate that autophagy activation may play a dual effect in APAP‐induced ALI models since autophagy can serve as a survival mechanism or a cell death mechanism in vivo.

### 3.2 Modulation of autophagy reduces D‐GalN/LPS‐induced ALI

Galanos et al proposed that coadministration of a certain dose of D‐GalN and endotoxin in animals leads to fulminant hepatic failure with extensive liver injury.^40^ The combination of D‐GalN and LPS is applied to induce the well‐defined experimental models with ALF. In addition, D‐GalN and endotoxin resulted in the cell death of a large number of hepatocytes in rodents by accelerating the progression of the caspase cascade and inducing DNA fragmentation as early as 5 hours after administration.^41^ On the other hand, an in vitro study showed that D‐GalN/LPS obviously aggravated hepatocyte injury by up‐regulating mitochondrial ROS and activating extracellular regulated protein kinase (ERK)1/2 and nuclear factor kappa B (NF‐κB) signalling pathways.^42^

The AMPK activator AICAR was shown to ameliorate D‐GalN/LPS‐induced ALF by down‐regulating the secretion of inflammatory factors, such as TNF‐α, IL‐1β and IL‐6 while increasing the expression of autophagy‐related proteins including Atg5, Beclin‐1, Atg7 and forkhead box O3A (Foxo3A).^43^ Treatment with FK866 or rapamycin before treatment with D‐GalN/LPS significantly ameliorated liver injury in ALF mice, as evidenced by decreased levels of ALT and aspartate aminotransferase (AST), and preserved liver histology via up‐regulation of autophagy and suppression of JNK and p62.^44^ Pre‐treatment with Wy‐14 643 significantly up‐regulated peroxisome proliferator–activated receptor alpha (PPAR‐α) expression and protected D‐GalN/LPS‐induced ALF mice against liver injury via activation of autophagy and suppression of inflammation, accompanied by inhibition of NF‐κB p65, JNK and ERK pathways.^45^ ACY1215, a histone deacetylase 6 inhibitor, helped to maintain normal liver histology and function in a D‐GalN/LPS‐induced ALF mouse model via up‐regulation of autophagy and inhibition of apoptosis and p62 expression, whereas the autophagy inhibitor 3‐MA aggravated liver tissue pathological and functional damage by accelerating the apoptotic process and reducing the mitochondrial membrane potential in ALF mice.^46^ It is worth noting that although autophagy is an important process that helps to clear damaged cellular contents to preserve liver function in D‐GalN/LPS‐treated animals, it also aggravates liver injury after activation of multiple cell death pathways. Phosphoinositide 3‐kinase (PI3K) agonist aggravated D‐GalN/LPS‐induced ALF in rats by increasing hepatic levels of PI3K, AKT, mTOR, Fas, Bax, p‐PI3K and p‐AKT while decreasing hepatic levels of Bcl‐2, LAMP2A and HSC70. However, the PI3K inhibitor effectively suppressed the progression of ALF by inhibiting the PI3K/AKT/mTOR signalling pathway.^47^

Intriguingly, several kinds of plant extracts that serve as effective antioxidant and anticancer agents have been proven to activate autophagy and reduce inflammation to inhibit the progression of D‐GalN/LPS‐induced liver injury. Treatment with tectorigenin or daphnetin protected against D‐GalN/LPS‐induced liver injury by activating autophagy and suppressing the secretion of inflammatory cytokines, including TNF‐α, IL‐6 and IL‐1β.^48^, ^49^ It was reported that tectorigenin also decreased Toll‐like receptor (TLR)‐4 expression and inhibited the activation of MAPK and NF‐κB pathways to attenuate liver injury.^48^ Furthermore, daphnetin notably inhibited liver dysfunction in D‐GalN/LPS‐induced ALF models by down‐regulating the levels of MDA, MPO, nitric oxide synthase (iNOS) and cyclooxygenase (COX)2 while up‐regulating the levels of GSH and SOD.^49^

In addition, mesenchymal stem cells (MSCs) and their derivatives are effective autophagy regulators in the inhibition of liver injury induced by D‐GalN/LPS. MSC transplantation prolonged the survival time of D‐GalN–induced ALF rats and decreased the levels of ALT and ammonia and the prothrombin time in ALF rats by up‐regulating autophagy and haem oxygenase 1 (HO‐1) expression but down‐regulating the release of inflammatory molecules such as TNF‐α, IL‐1β, IL‐6 and IL‐12p40 in a PI3K/AKT‐mediated manner.^50^ MSC‐derived exosomes reversed liver injury in D‐GalN/LPS‐treated primary hepatocytes by up‐regulating LC3, Beclin‐1 and autophagosome formation. Moreover, these exosomes increased the expression of the antiapoptotic protein Bcl‐2 and decreased the expression of the proapoptotic proteins Bax and cleaved caspase‐3.^51^

### 3.3 Modulation of autophagy reduces ConA‐induced ALI

It is widely accepted that ConA‐treated animal models generally serve as an experimental model of acute immune hepatitis (AIH). ConA is reported to induce immune hepatitis by promoting hepatocyte apoptosis and inhibiting T‐cell function after induction of autophagic cell death in hepatocytes and liver endothelial cells in vitro and in vivo.^52^, ^53^ The frequency of hepatic and peripheral accumulation of mature conventional dendritic cells (DCs) was reported to be positively correlated with AIH severity. Bone marrow–derived DCs from AIH mice exhibited higher expression of inflammatory factors, autophagy‐related proteins and autophagosomes than those from wild‐type mice.^54^

However, most studies indicated that a reduction in autophagy would contribute to the attenuation of ConA‐induced liver injury since ConA generally triggers both autophagic cell death and apoptotic cell death. Methylprednisolone, a kind of glucocorticoid, is proven to be the most effective treatment for AIH. Methylprednisolone effectively inhibited apoptosis and autophagy in hepatocytes from ConA‐induced AIH through down‐regulation of inflammatory reactions and activation of the AKT/mTOR signalling pathway.^55^ Necrostatin‐1 effectively preserved liver functions and maintained normal histopathology in ConA‐induced ALI models by suppressing the secretion of inflammatory cytokines (TNF‐α, IFN‐γ, IL‐2, IL‐6 and cobalt ion binding protein) and the formation of autophagosomes.^56^ Administration of 15d‐PGJ2 attenuated ConA‐induced ALI by inhibiting autophagy flux and mitochondrial ROS and the release of inflammatory factors (TNF‐α and IL‐1β) while up‐regulating the expression of the antioxidative stress factor HO‐1 in mouse models.^57^

Oleanolic acid injection significantly preserved liver function, improved the expression of PPAR‐*α* and inhibited the phosphorylation of JNK to attenuate liver apoptosis and autophagy.^58^ Pre‐treatment with astaxanthin or shikonin before ConA injection prevented ALI, as demonstrated by decreased serum levels of liver enzymes and inflammatory cytokines via down‐regulation of JNK/p‐JNK–mediated apoptosis and autophagy.^59^, ^60^ Preconditioning with quercetin, a member of the flavonoid family, also significantly decreased the expression levels of liver enzymes and inflammatory cytokines. Quercetin down‐regulated the expression of Bax/Bcl‐2 and caspase‐9 to attenuate apoptosis and decreased the expression of LC3 and p62 to attenuate autophagy in ConA‐induced AIH models.^61^ In addition, preconditioning with another flavonoid compound, isorhamnetin, remarkably decreased the serum levels of liver enzymes and inflammatory cytokines and improved pathological damage in ConA‐induced ALF mice by inhibiting apoptosis and autophagy via the P38/PPAR‐α pathway.^62^ Pre‐treatment with fucosterol, which is isolated from the brown alga *Eisenia bicyclis*, preserved liver functions and attenuated liver necrosis and apoptosis by decreasing the release of TNF‐α, IL‐6, and IL‐1β, down‐regulating Bax/Bcl‐2 and inhibiting P38 MAPK/PPARγ/NF‐κB signalling.^63^ Pre‐treatment or cotreatment with salidroside, a glycoside extract isolated from *Rhodiola rosea* L., is effective in reducing the expression of liver enzymes and attenuating pathological damage via suppression of inflammation and the PI3K/AKT signalling pathway to inhibit apoptosis and autophagy in ConA‐treated mice.^64^

As ConA was demonstrated to be a potent autophagy inducer via a mitochondria‐mediated pathway,^65^ most studies have been effective in attenuating ConA‐induced ALI via reduction in autophagy. However, we recommend expanding the related studies on autophagy regulation and liver injury recovery in ConA‐treated animal models because there is also evidence indicating that autophagy up‐regulation contributes to liver protection. For example, treatment with FK866 or rapamycin before treatment with ConA ameliorated liver injury in ALF mice via up‐regulation of autophagy and suppression of the JNK signalling pathway.^44^

### 3.4 Modulation of autophagy reduces CCl_4_‐induced ALI

CCl_4_ is reported to induce high levels of oxidative stress, inflammation, necroptosis and apoptosis in liver tissue by up‐regulating hypoxia‐inducible transcription factor‐1α (HIF‐1α) expression and activating the TLR4/NF‐κB pathway.^66^ In CCl_4_‐induced ALF models, the expression of KLF6 is activated to enhance autophagy and liver regeneration through transcriptional induction of Atg7 and Beclin‐1 in a p53‐dependent manner.^33^ Another study indicated that the caspase‐9 inhibitor z‐LEHD‐FMK aggravated CCl_4_‐induced ALI in HepG2 cells, AML12 cells and mouse models via down‐regulation of cytoprotective autophagy, while up‐regulation of HIF‐1α resulted in oxidative stress and TLR4/NF‐κB–mediated inflammation.^66^ Pre‐treatment with genipin has been proven to induce the conversion of LC3 and inhibit p62 accumulation in vivo, subsequently attenuating CCl_4_‐induced ALI via up‐regulation of autophagic flux and down‐regulation of NF‐κB‐ and STAT3‐dependent inflammation.^67^ Although autophagy generally serves as a protective mechanism in CCl_4_‐induced ALI in vitro and in vivo, Dai et al argued that chloroquine pre‐treatment attenuated the secretion of high‐mobility group box 1 (HMGB1)–mediated inflammatory factors such as IL‐6 and TNF‐α and inhibited autophagy to attenuate CCl_4_‐induced ALI. In addition, chloroquine pre‐treatment also inhibited apoptosis progression by down‐regulating NF‐κB, p53, the ratio of Bax/Bcl‐2, and caspase‐3 in liver tissue.^68^

### 3.5 Genetic modification of autophagy has dual effects on chemically induced ALI

Although knockdown of autophagy‐related key factors in some models aggravated chemically induced liver injury, it also provided strong resistance to liver damage. Knockout of Atg7 in mice aggravated APAP‐induced liver injury via activation of caspase‐3, caspase‐7 and JNK, which resulted in the accumulation of mitochondrial membrane depolarization, mitochondrial ROS and hepatocyte apoptosis.^69^ Knockout of Atg7 promoted the activation of macrophages, release of inflammatory factors, accumulation of dysfunctional mitochondria and disruption of ROS degradation via up‐regulation of p38/MAPK and NF‐κB pathways in ConA‐induced ALI.^70^ Similarly, deletion of eva‐1 homolog A (Eva1a) inhibited autophagy progression and aggravated liver injury, as evidenced by up‐regulation of aminotransferases, MPO, inflammatory cytokines and mitochondrial injury in D‐GalN/LPS‐induced ALF mice.^71^

Specific deletion of Atg5 in mice resulted in constitutive activation of the NRF2 signalling pathway to up‐regulate drug detoxification and GSH synthesis, subsequently promoting hepatocyte proliferation and enabling mice to resist APAP overdose–induced injury.^72^ On the other hand, Sun et al demonstrated that although targeting factors upstream of autophagy did not alter autophagy levels, the regulation protected against liver injury via other mechanisms in APAP‐induced liver injury models. Although knockout of Ulk1/2 did not alter the autophagic activity of hepatocytes in mice upon overnight fasting, these mice showed strong resistance to APAP‐induced ALI via activation of JNK signalling both in vivo and in vitro.^73^ Deletion of apoptosis‐stimulating protein 2 of p53 (ASPP2) in mice protected them from CCl_4_‐induced ALI via activation of autophagy and reduction of cellular inflammation and apoptosis, as shown by decreased ALT and AST levels and reduced hepatic tissue haemorrhage and necrosis.^74^ According to these studies, we predict that genetic modifications will repair liver injury by regulating autophagy.

## 4 CONCLUSIONS

The formation of autophagosomes and packaging of cytoplasm in liver cells participate in breaking down enclosed cargo inside lysosomes to replenish new energy sources and other components. Autophagy generally serves as a cell survival mechanism in stressful microenvironments, but it also serves as a destructive mechanism that results in cell death in vitro and in vivo. According to our summary, there exists a general regulation of autophagic flux in different ALI animal models. A large number of studies have shown that the activation of autophagy contributes to protection from ALI induced by APAP, D‐Gal/LPS or CCl_4_, while the inhibition of autophagy protects from liver injury in ConA‐induced AIH. It is worth noting that various drugs or plant extracts are effective for the regulation of autophagy to protect against ALI induced by chemicals via different mechanisms. Another important issue is the specific factor that switches autophagy from a survival mechanism to autophagic cell death. Although genetic modifications in animal models targeting autophagy regulation help to protect against ALI, the safety and clinical utility of genetic modifications are uncertain. After clarification of how to selectively control the dual effects of hepatic autophagy in various animals, we are more confident in the ability to improve the prognosis of ALI by modulating autophagy.

## CONFLICT OF INTEREST

The authors declare no competing financial interests.

## AUTHOR CONTRIBUTION

**Chenxia Hu:** Project administration (lead); Resources (lead); Writing‐original draft (lead). **Lingfei Zhao:** Project administration (equal); Resources (equal); Writing‐original draft (equal). **Miaoda Shen:** Writing‐review & editing (lead). **Zhongwen Wu:** Writing‐review & editing (equal). **Lanjuan Li:** Conceptualization (equal).

## Data Availability

Not applicable.
